# Establishment and Characterization of New Canine and Feline Osteosarcoma Primary Cell Lines

**DOI:** 10.3390/vetsci3020009

**Published:** 2016-06-01

**Authors:** Florian R. L. Meyer, Ingrid Walter

**Affiliations:** Department of Pathobiology, Institute of Anatomy, Histology and Embryology, University of Veterinary Medicine, Veterinaerplatz 1, Vienna 1210, Austria; florian.r.l.meyer@gmail.com

**Keywords:** osteosarcoma, cell culture, dog, cat

## Abstract

Osteosarcomas are the most abundant form of bone malignancies in multiple species. Canine osteosarcomas are considered a valuable model for human osteosarcomas because of their similar features. Feline osteosarcomas, on the other hand, are rarely studied but have interesting characteristics, such as a better survival prognosis than dogs or humans, and less likelihood of metastasis. To enable experimental approaches to study these differences we have established five new canine osteosarcoma cell lines out of three tumors, COS_1186h, COS_1186w, COS_1189, and COS_1220, one osteosarcoma-derived lung metastasis, COS_1033, and two new feline osteosarcoma cell lines, FOS_1077 and FOS_1140. Their osteogenic and neoplastic origin, as well as their potential to produce calcified structures, was determined by the markers osteocalcin, osteonectin, tissue unspecific alkaline phosphatase, p53, cytokeratin, vimentin, and alizarin red. The newly developed cell lines retained most of their markers *in vitro* but only spontaneously formed spheroids produced by COS_1189 showed calcification *in vitro*.

## 1. Introduction

Tumors are one of the major diseases in both human and companion animals with an annual incidence rate of about 182/100,000 in humans [1] 381/100,000 in dogs and 156/100,000 of cats [2]. When comparing numerical data between human and veterinary patients it has to be considered that a number of unrecorded cases are present in animal patients, in particular in the feline species. It is, therefore, of major clinical importance to investigate issues such as tumor progression and metastasis behavior, the main reason for tumor related death, and to test tumor cells for drug response. For ethical reasons, *in vitro* methods are preferable whenever possible over experiments on living animals. A suitable method for experimental approaches, especially drug tests in cancer research, is the use of cell cultures. Due to of the natural immortality of tumor cells, they retain their *in vivo* features better than normal cells which have to be artificially immortalized to be used for longer periods of time [3,4,5]. 

Osteosarcoma is the most common neoplasm of the bone in human patients, cats, and dogs, and it is generally assumed that it derives from osteoblastic cells, but now pluripotent stem cells have also been under discussion as a source of neoplastic cells [6,7,8]. Due to the many similarities between human and canine osteosarcomas, the latter is considered a valuable model organism for this disease [9,10]. These similarities include the sites most often affected, a predominance of male sex, and the high likelihood of metastasis development, preferably to the lung [10]. Due to the generally late diagnosis of the disease, micrometastases are often already present at the time of diagnosis [11]. However, there are also noteworthy differences between osteosarcomas of the two species, like the age of onset; generally, adolescence in humans—although there is a notable second peak in patients >60 years [12]—and middle-aged to older dogs are affected [13]. It also seems that human osteosarcoma is caused by growth during adolescence [14,15], while the older age of onset in canines has been supposed to be induced by mechanical forces, such as (micro-) fractures [16].

A number of cell lines have successfully been cultivated and characterized from human [11,16,17,18,19] and canine [20,21] osteosarcomas. While a large number of human osteosarcoma cell lines are commercially available, like HOS [22], U2 OS [23], Saos-2 [24], or MG-63 [25], only the canine osteosarcoma cell line D-17 (CCL-183) [26] is accessible for scientists. Feline osteosarcomas, on the other hand, were rarely studied, and to our knowledge this work presents the first establishment and description of a feline osteosarcoma cell line. Despite this, we believe feline osteosarcoma to be a valuable model in understanding osteosarcoma tumor mechanisms. While feline osteosarcomas have a strong histological similarity to human and canine osteosarcomas [27], their behavior in tumor progression, especially the much lower rate of metastases [28], stands opposed to their human and canine counterparts.

The aim of the present study was the isolation and cultivation of primary cells from different types of canine and feline osteosarcomas. The cell cultures were tested for bone tumor markers as determined before, such as tissue unspecific alkaline phosphatase [29,30], osteocalcin [31], and osteonectin [19,20,32,33]. The deposition of calcified structures was assessed by alizarin red staining [34]. Cultivated osteosarcoma cells were further characterized immunohistochemically for vimentin [20,32,33] and cytokeratin intermediate filaments [32] and the tumor-suppressor p53 [33]. All of the above-mentioned factors were comparatively stained on formalin-fixed paraffin-embedded (FFPE) sections of the corresponding tumor tissue. Furthermore, the origin of cells from the respective tumor was verified by DNA fingerprinting.

## 2. Experimental Section

### 2.1. Animals

Osteosarcoma tumor samples from dogs (*n* = 4) and cats (*n* = 2) were collected after therapeutic limb amputation or euthanasia. The study was approved by the Ethical and Animal Welfare Committee of the University of Veterinary Medicine (15 December 2014) and conducted according to the guidelines of the local ethical committee. Animal data and tumor subtypes are reported in Table 1. Tumor tissues were transferred under sterile conditions to the VetBiobank of the VetCore Facility for Research of the University of Veterinary Medicine, Vienna. Tumors were dissected and aliquots of tumor tissue were formaldehyde-fixed and paraffin-embedded or shock frozen in liquid nitrogen with and without RNAlater (Life Technologies, Vienna, Austria) for RNA and DNA analysis. Before embedding in paraffin, strongly calcified samples were decalcified in 8% EDTA before the embedding process. Further parts of the tumors were used for cell culture experiments. 

### 2.2. Cell Culture

Pieces of fresh tumor tissue were cut into cubes of about 1 mm^3^. The pieces were washed three times in DPBS (Sigma Aldrich, Vienna, Austria) to remove erythrocytes and then transferred to individual wells of a four-well plate (Thermo Fischer Scientific Vienna, Austria) with either DMEM (Sigma Aldrich) or Q286 medium (GE-Healthcare, Munich, Germany), supplemented with 10% fetal calf serum (FCS, Sigma-Aldrich), 625 pg/100 mL Amphotericin B (Bio and Sell, Feucht, Germany), 2 nm L-glutamine (Biochrom, Berlin, Germany), and 1% Pen/Strep/Fungi Mix (10,000 U Penicilin; 10 mg Streptomycin; 25 µg Amphotericin B/mL, Bio and Sell), or the complete osteoblast growth medium (OGM BulletKit; Szabo Scandic, Vienna, Austria). Cells were incubated at 37 °C and 5% CO_2_. The first medium change was done after 24 h; after that, medium was changed in two day intervals. When cells started to grow out from the tissue pieces and attached to the plastic surface of the cell culture dish, the tissue pieces were detached mechanically from the plastic surface with a pipette tip to generate new spawning points within the well. At near-confluency, cells were trypsinized (Trypsin/EDTA solution, Merck, Darmstadt, Germany) and transferred to six-well plates (Sarstedt, Nümbrecht, Germany). After reaching near-confluency in the six-well plate, cells were transferred to 25 cm^2^ cell culture flasks (Sarstedt) and expanded in 25 cm^2^ and 75 cm^2^ cell culture flasks (Sarstedt). Cells were frozen as soon as two 25-cm^2^ cell culture flasks reached 80% confluency each, by resuspending trypsinized cells in a 1.5 M DMSO supplemented medium and using a CoolCell (Biozym, Vienna, Austria) to bring samples to −80 °C at a controlled cooling rate before storing them in liquid nitrogen.

For histological purposes, cells were scraped off a 25 cm^2^ flask, at about 80% confluency and fixed in 4% buffered formaldehyde overnight. The cells were then pelleted by centrifugation (5000 g for 2 min) and the cell pellets pre-embedded in Histogel™ (Thermo Fischer Scientific, Vienna, Austria) and, subsequently, paraffin embedded. 

### 2.3. Histology

FFPE samples from original tumor tissue and pellets of cell cultures were used for histological analysis. Sections measuring 3 µm were mounted on glutaraldehyde-activated 3-aminopropyl-triethoxysilane (APES)-coated glass slides.

### 2.4. Immunohistochemistry

A summary of the techniques and antibodies used in immunohistochemistry can be found in Table 2. For tissue unspecific alkaline phosphatase endogenous peroxidases were blocked using 0.6% H_2_O_2_ in 80% methanol. Epitope retrieval for tissue and cell pellets was done by steaming or microwaving as indicated in Table 2. 1.5% normal goat serum (Dako) was used for protein blocking. Sections/coverslip cultures were incubated with the respective primary antibody over night at 4 °C. For osteonectin, osteocalcin, vimentin, and cytokeratin, Alexa Fluor 488 goat-anti-mouse IgG (H + L) highly cross-adsorbed (Life Technologies; dilution 1:100 in PBS) was used as the secondary antibody. DAPI (Sigma Aldrich) was used for nuclear staining. The BrightVision Poly-HRP-anti-rabbit (ImmunoLogic, Duiven, Netherlands) was used as the secondary antibody for alkaline phosphatase and p53 staining, and slides were counterstained with haematoxylin. Sections prepared without primary antibody served as negative controls. For tissue unspecific alkaline phosphatase, osteocalcin canine phalanx and joint sections and feline femur sections were used as controls. Canine uterus was used as a control for vimentin and cytokeratin. 

To determine a potential calcification of cultured osteosarcoma cells, slides of cell culture pellets and tumor were stained for 1 h in alizarin red (Morphisto, Frankfurt, Germany) pH 9.0 and 5 min alizarin red pH 7.0 (Morphisto).

### 2.5. PCR

To verify the presence of ostecalcin (*BGLAP*), osteonectin (*SPARC*), and tissue unspecific alkaline phosphatase (*ALPL*), an mRNA amplicon dissociation assay was used. RNA was isolated from cells and corresponding tumours using the miRNeasy Mini kit (Qiagen, Hilden, Germany) and the RNeasy Fibrous Tissue Mini kit (Qiagen) according to the manufacturer’s protocol, respectively. The amount of 500 ng RNA was transcribed into cDNA using the High-Capacity cDNA Reverse Transcription Kit (Applied Biosystems, Foster City, CA, USA). The reaction volume of 20 µl contained 2 µL 10 × RT Buffer, 0.8 µL 25 × dNTP Mix (100 mM of each dNTP), 2 µL 10 × RT random primers, 50 U multiscribe reverse transcriptase and 14.2 µL of RNase-free H_2_O containing the 500 ng of RNA. The reaction was incubated for 10 min at 25 °C to initiate primer binding and was followed by 2 h at 37°C for transcription. The reaction was stopped by heating to 85 °C for 5 s.

For PCR 5 ng of cDNA was mixed with 1 µL buffer BD (Solis Biodyne, Tartu, Estonia) 3.5 mM MgCl_2_, 0.2 mM of each dNTP (Solis Biodyne) 0.4 × EvaGreen (Biotium, Hayward, CA, USA), 200 nM of each primer (Sigma-Aldrich) 0.5 unit hot-start Taq DNA polymerase (HOT FIREPol^®^ DNA polymerase; Solis Biodyne) in a 10 µL reaction volume. Cycling conditions consisted of a hot start at 95 °C for 15 min followed by 40 cycles of denaturation for 15 s at 95 °C, annealing for 20 s at 60 °C and elongation for 20 s at 72 °C. For analysis a melting curve analysis was performed after the PCR, were the reaction mixture was heated from 60 °C to 95 °C at a ramp rate of 0.03 °C/s acquiring 20 data points per °C (primer sequences are given in Table 3).

All reactions were performed as technical duplicates with the inclusion of combined RT-controls and negative controls also performed in duplicate on a Light Cycler 480 (Roche Diagnostics, Vienna, Austria).

### 2.6. DNA-Fingerprint Assay

DNA of original fresh frozen tumor aliquots and primary cell cultures (passages used were: COS_1033: P5, FOS_1077: P12, COS_1140: P15, COS_1186h: P4, COS_1186w: P7, COS_1189: P23, COS_1220: P6) were isolated using the Nucleo Spin Tissue Kit (Macherey-Nagel, Düren, Germany). In brief, ~20 ng of tissue or a pellet of 10^5^ to 10^6^ cells was used as starting material. Tumor tissue samples were homogenized in T1 buffer (Macherey-Nagel) using zirconium beads (PEQLAB Biotechnologie, Erlangen, Germany) in the MagNA Lyser (Roche Diagnostics) for 2 times 20 s at 6500 rpm and were cooled on ice in between. Cultured tumour cells were homogenized by vortexing vigorously in T1 buffer (Macherey-Nagel). Homogenates were than lysed with proteinase K (Macherey-Nagel) for 15 min and DNA isolated as described in the manufacturers protocol.

DNA was sent to Laboklin (Bad Kissingen, Germany) for DNA fingerprinting determining 18/16 marker regions for dogs or cats, respectively [35].

## 3. Results

**Figure 1:**
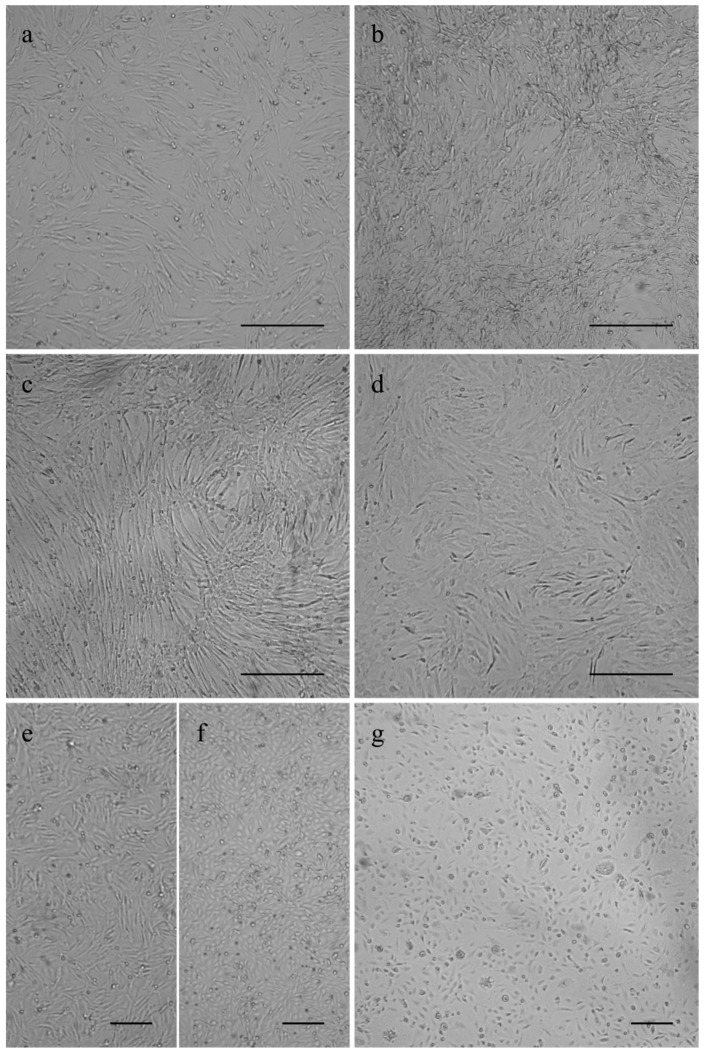
Phase contrast images showing growth morphology of (**a**) COS_1033 at P4; (**b**) COS_1189 at P7; (**c**) COS_1220 at P4; (**d**) FOS_1140 at P4; (**e**) COS_1186w at P3; (**f**) COS_1186h at P4; and (**g**) FOS_1077 at P4. Scale bars represent 100 µm for (**a**–**d**) and 200 µm for (**e**–**g**).

The canine osteosarcoma cells COS_1033, COS_1189 and COS_1220 were successfully grown to the passages 8, 25, and 6, respectively. The canine tumor 1186 macroscopically showed a distinct separation in a hard, bone- or cartilage-like area and a soft tissue-like area. Separate tissue cultures were established from both tumor parts and labelled as COS_1186h for the hard part and COS_1186w for the soft part, respectively. COS_1186h was grown to passage 4 and COS_1186w was grown to passage 7. The feline osteosarcoma cell lines were grown up to passage 21 for FOS_1077 and 19 for FOS_1140. Cells did not change their growth characteristics or proliferation within the stated number of passages. Most canine cell lines, namely COS_1033, COS_1186w, COS_1189, and COS_1220, showed a fibroblast-like morphology when grown as monolayers on plastic cell culture dishes or glass surfaces irrespective from the tumor subtype (Figure 1). In contrast, the “sister cell line” of COS_1186w (obtained from the same tumor), COS_1186h, showed a cobblestone epithelial-like morphology (Figure 1). Feline osteosarcoma cell lines (FOS_1077 and FOS_1140) had a mixed morphology of fibroblast-like cells and osteoblast like cells (Figure 1).

COS_1189 showed a weak attachment to plastic surfaces and once a continuous layer of cells was formed (~70%–80% confluency), even light agitation of the cell culture dish led to the detachment of sheets of cells which formed spheroids spontaneously.

**Figure 2:**
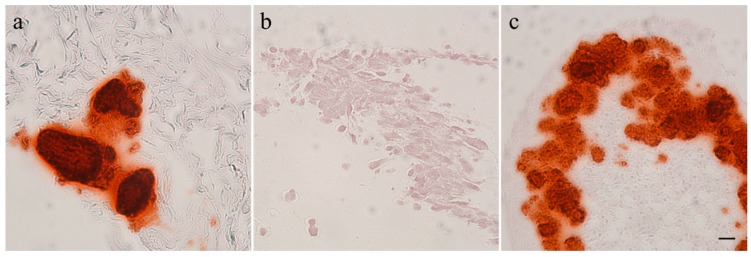
Alizarin red staining of (**a**) a tumor section of 1189; (**b**) a monolayer cell culture sample of COS_1189; and (**c**) a spheroid of COS_1189. Scale bar represents 20 µm.

Calcification of the tumors and tumor cell cultures was determined by alizarin red staining. Feline tumor 1077 and canine tumor 1186 showed large areas of calcification; in the latter both in the macroscopically hard and soft areas. The three other canine osteosarcomas showed only few calcified spots and no signs of calcification were found in feline osteosarcoma 1140. It has to be noted that the amount of calcification was observed to be very heterogeneous within the tumors. All canine and feline cell cultures were alizarin red negative after two (COS_1033), three (COS_1186w and COS_1220), four (COS_1186h), five (FOS_1077), seven (FOS_1140), or 11 (COS_1189) days of culture. The spontaneously-developing spheroids, as observed in COS_1189 cultures, showed strong calcification after three days in culture, as demonstrated by alizarin red staining (Figure 2), but the center of the spheroids also showed signs of necrosis.

### 3.1. Immunohistochemistry

**Figure 3:**
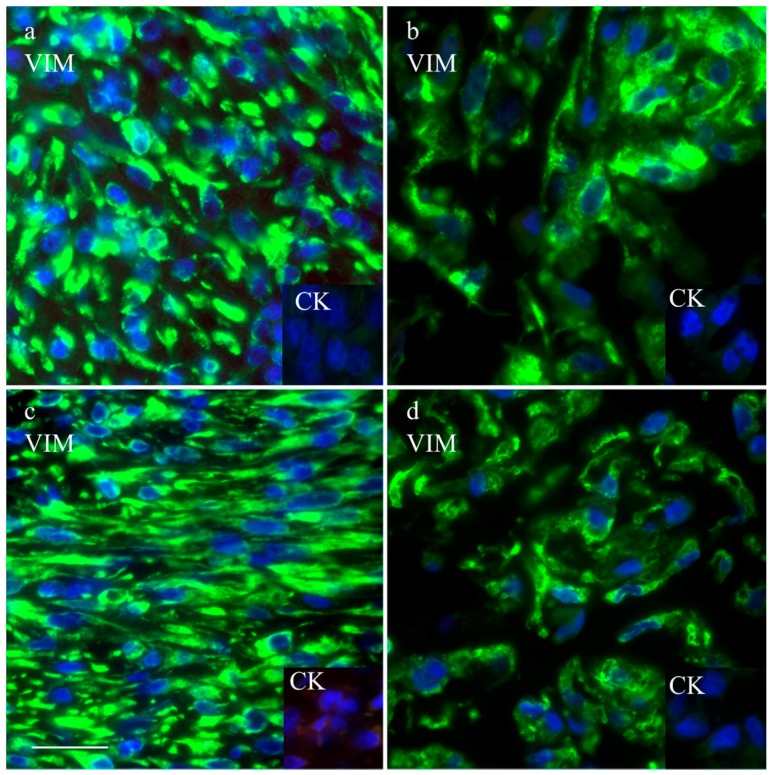
Immunofluorescence staining with anti-vimentin and anti-cytokeratin (inserts) of the canine tumor 1033 (**a**) and the resulting cell culture (**b**) as well as the feline tumor 1077 (**c**) and its resulting cell culture (**d**). Scale bar represents 25 µm.

**Figure 4:**
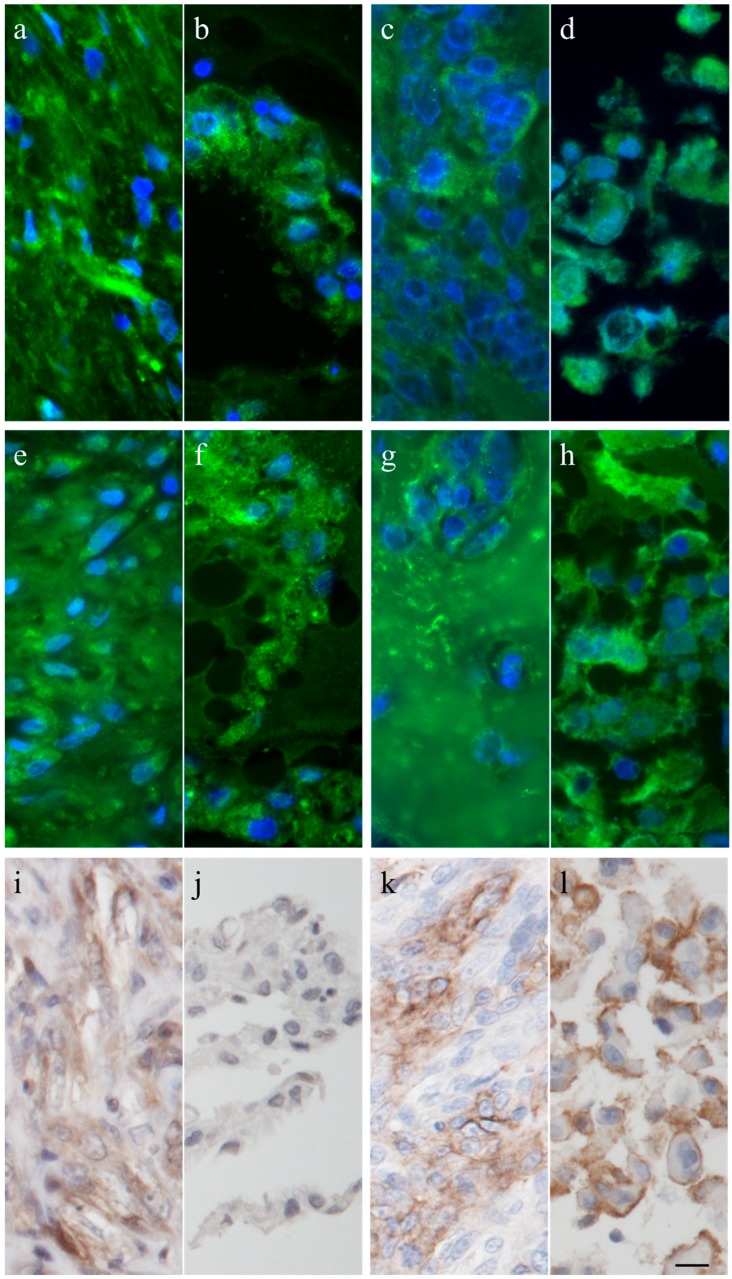
Immunhistochemistry and immunofluorescence of canine tumor 1220 (**a**,**e**,**i**) and its cell line (**b**,**f**,**j**), and the feline tumor 1077 (**c**,**g**,**k**) and its cell line (**d**,**h**,**l**) showing osteonectin (**a**–**d**), osteocalcin (**e**–**h**), and tissue unspecific alkaline phosphatase (**i**–**l**). Scale bar represents 10 µm.

**Figure 5:**
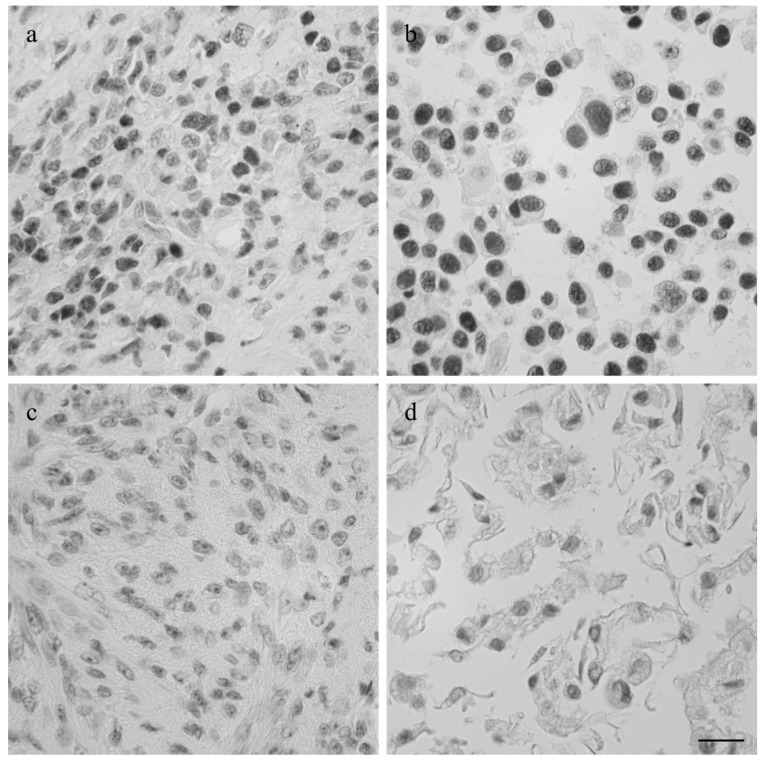
Imunhistological staining of p53 in the canine tumor 1189 (**a**) and feline osteosarcoma 1140 (**c**), and the cell lines COS_1189h (**b**) and FOS_1140 (**d**) derived from these tumors. Scale bar represents 20 µm.

The intermediate filament cytoskeleton of the cultivated cells, irrespective of their morphology, was composed of vimentin as expected for mesenchymal cells and as also seen in the corresponding histological tumor sections (Figure 3). Cytokeratin positive tumor cells were never observed, neither in the original tumors nor in the cultivated tumor cells (Figure 3). Immunohistochemistry demonstrated osteonectin and osteocalcin in every examined tumor, albeit in different intensity levels (Figure 4). Osteonectin was less abundant in cat tumors compared to their canine counterparts. The expression of these proteins was also detectable in the respective corresponding cell cultures of feline and canine osteosarcomas (Figure 4). All feline and canine osteosarcomas were positive for tissue unspecific alkaline phosphatase. This marker remained unchanged in the corresponding primary cell cultures with the exception of one canine cell line (COS_1220) where the original tumor was positive, but no expression was observed in the cell culture. The distribution of the signal was cytoplasmic, membrane bound or nuclear (Figure 4). p53 showed no staining in cats, whether the tumor itself or the corresponding cell culture, but was present in nuclei of all canine cell lines (Figure 5). It has to be noted that the tumor 1189 showed only very few stained cells, while the resulting cell line COS_1189 was clearly positive.

### 3.2. PCR

By means of PCR, the presence of mRNA of tissue unspecific alkaline phosphatase (*ALPL*) and osteonectin (*SPARC*) was detected positively in all tested feline and canine tissue and cell culture samples, except for the cell culture samples of COS_1220 which lost ALPL expression, mirroring the results obtained by immunohistochemistry. However, we failed to amplify osteocalcin (*BGLAP*) mRNA in feline and canine samples (A list of the results is supplied in Supplementary Table S1). 

### 3.3. DNA Fingerprint Assay

Microsatellite analysis of the established primary cell lines confirmed their origin from the corresponding tumor. In feline samples 16 markers were tested. All markers were identical between tumor tissue and cells in culture. In dogs, 18 markers were tested. COS_1033 and COS_1186h cells and tumor were completely identical in their marker setup, although one marker failed to amplify in the latter, both in tumor and in the cell line. COS_1220 showed one allele changed in a single marker, COS_1189 had alterations in two alleles. Three markers differed between COS_1186w and the tumor, although one marker failed to amplify in the tumor sample, but not in the cell line. A complete list of the obtained microsatellite data as first published in [35] is supplied in Supplementary Tables S2 and S3. These lists can be helpful in future experiments to exclude cross contaminations. 

## 4. Discussion

Osteosarcomas are the most common bone neoplasms in humans [12], dogs [36], and cats [37]. Osteosarcomas in dogs are considered a valuable model for human osteosarcomas, because of the similar behavior of these two diseases concerning tumor progression and metastasis development in the lung [9,10,38]. In contrast, little research has been done on feline osteosarcomas, but current data points to a much less severe progress with less likelihood of metastasis in this species, although the histological properties of the primary tumor are very similar to its human and canine counterpart [27,26]. Osteosarcoma cell lines have been applied in many fields of research such as drug development [39,40], drug resistance development [41], and basic tumor research [42,43]. Mostly, these studies have been focused on human osteosarcoma cell lines [39,41,42,43,44], but also osteosarcomas of rat [45], mouse [46], and dog [40,43,46] were used for investigations. The use of canine and feline osteosarcoma cell cultures would be of particular interest allowing scientists to investigate the biological differences of these two species as reflected by the diverse metastatic behavior of these otherwise very similar tumors. Today, no feline osteosarcoma cell line is available or described in the literature to investigate their specificities and to use them in experimental applications. 

We, therefore, developed five canine, and two feline, primary cell lines out of three canine osteosarcomas, one canine osteosarcoma-derived lung metastasis, and two feline osteosarcomas. Tumor cells attached to the plastic surface in a standard cell culture environment and were readily grown in provided media supplemented with fetal calf serum. Initially, two standard media (DMEM and Q286) and a medium specialized for the rearing of osteoblast cells (OGM), were chosen to initiate cell culture, however no preference to a single medium by a certain cell line was apparent. The applied method of establishment of the primary cell lines was robust and repeatable. There are two different main approaches for bringing osteosarcoma cells into culture, either by digesting the tumor matrix by collagenases [20,21] or by directly bringing minced pieces of osteosarcoma into cell culture [22,32]. While the digesting approach leads to a higher number of cells more quickly, and might be beneficial if cells need a high density for survival, it might damage the cells [47]. We have chosen the mincing approach because we are convinced that, preferably, tumor cells will grow out of the minced pieces, reducing contamination from non-tumor cells in the early passages. 

Canine cell lines had a strong tendency to produce a fibroblast like morphology in cell cultures COS_1033, COS_1186w, COS_1189, and COS_1220, irrespective of the subtype. Only COS_1186h kept the osteoblastic morphology of the originally osteoblastic osteosarcoma in cell culture. Both feline cell lines showed a mixed phenotype of osteoblastic and fibroblastic cells. The differences in the morphology of the two sister cell lines COS_1186w and COS_1186h confirms the well-known inter-tumoral heterogeneity of the osteosarcoma [48,49]. Two tumor cell lines with basically the same genetic layout, but different morphological features, offers interesting prospects to further the understanding of tumor development, progression and drug response. 

COS_1189 cells showed a strong tendency to adhere more strongly to each other than to the plastic surface of the dishes they were grown in. Depending on the confluency of cells and size of the dish, spheroids could be produced by simply shaking the dish. If done cautiously all cells tended to aggregate to one spheroid, but more likely two to four spheroids developed. Due to these large assembled spheroids, nutritional supply for cells in the middle was limited and these cells showed signs of degradation at the time of collection. In contrast to the cells grown as monolayers, cells within the spheroid deposited calcified osteoid-like areas, resembling the *in vivo* situation in osteosarcomas. We assume that a three-dimensional culture model would be preferable to better mimic the *in vivo* situation.

The established cell lines retained most of the characteristics of the tumors they derived from. We used the bone markers osteonectin [20,32,46], osteocalcin [31], and tissue unspecific alkaline phosphatase [29,30] at the protein and mRNA levels, confirming the origin of the cells that were obtained in cultures. These findings are in accordance with previously-established osteosarcoma cell lines that were positive for osteonectin, osteocalcin, and tissue unspecific alkaline phosphatase [30].

Alkaline phosphatase activities were found in other canine osteosarcoma cell lines [18,20,30] but also described as low [21]. Legare *et al.* [30] reported that most tested canine cell lines lost alkaline phosphatase immunoreactivity during culture time, however, only the very aggressive “Abrams” cell line consistently stained positive. In human osteosarcoma cells, tissue unspecific alkaline phosphatase activity was dependent on cell density [19], therefore, this condition has to be considered when comparing data about alkaline phosphatase. No data about alkaline phosphatase in feline osteosarcoma tumors or cell lines exist until now. Although higher serum levels of alkaline phosphatase have been correlated with shorter survival time [38], our results do not support tissue unspecific alkaline phosphatase as a distinguishing marker for the aggressive canine osteosarcoma from the low metastasizing feline osteosarcoma, as samples of both species were positive for tissue unspecific alkaline phosphatase, albeit the sampled tumor section of osteosarcoma 1189 showed only weak staining.

Osteocalcin, a small peptide hormone produced by osteoblasts, was used to verify the bony origin of our new cell lines. Immunhistochemistry was positive for osteocalcin in all tested samples, however, RT-PCR failed in all tested cases. Bioinformatically, osteocalcin is highly conserved and only one splice variant and one polycistronic mRNA containing BGLAP (PMF1-BGLAP) is known in the best-studied species, humans, and forms of these two transcripts are also predicted in cats and dogs. We are unsure why primers for this gene failed. Additionally, while all positive controls failed for this gene we do not consider it as evidence against the osteocalcin-antibody specificity. 

Osteonectin is one of the most abundant proteins in the bone. It was present in all tested tumors and cell cultures, but its staining was notably weaker in feline tumors than it was canine osteosarcomas. Osteonectin is known to have tumor-progressing or -suppressing properties, depending on the surrounding cellular environment, and seems to be a negative prognostic marker in human osteosarcoma patients [50], indicating a valuable candidate gene that might influence the progression of osteosarcoma.

All established canine and feline cell cultures expressed vimentin irrespective of growth behavior but were negative for cytokeratin. This result is in contrast with the data from Nagamine *et al.* [51] who found canine osteosarcomas of various subtypes positive for cytokeratin and vimentin. 

We tested calcification of tumor tissue and cell lines by alizarin red staining. All tumor samples showed calcified areas, except one feline tumor (1140), but no cell monolayer showed an alizarin positive staining. However, the spontaneous spheroids developed by COS_1189 showed alizarin red staining. While Mohseny *et al.* [33] showed that monolayers of HOS cells showed strong calcification, Martins-Neves *et al.* [34] showed only alizarin-positive spheroids, although both were grown in the media RPMI 1640. Prolonged sustaining the cells as a monolayer or differences in cell culture maintenances might influence calcification and explain these differences.

p53 is a transcription factor that is involved in cell cycle control [52] and it is known that disruption of p53 function strongly correlates with tumorigenesis [53]. In all our established canine cell lines p53 was present, as in their corresponding tumors. This is in accordance with the results of Bongiovanni *et al.* [54] who found p53 in canine appendicular osteosarcoma by means of immunohistochemistry. Moreover, p53 was correlated with the histological grade, as grade III tumors were 100% positive in canine osteosarcoma, and found to be a negative prognostic marker [54]. Interestingly, p53 was negative in both investigated feline tumors and the corresponding cell lines. It has been reported before that only about 50% of feline osteosarcomas express p53 [55] and that feline tumors tend to delete p53 [56]. Positivity might also be correlated to a certain subtype of osteosarcoma, however, tumor type was not stated in the report of Nasir *et al.* [55] and the number of cases investigated was low. Therefore, this hypothesis has to be verified on a statistically significant number of feline osteosarcomas. Further studies are needed to determine if p53 is involved in the characteristically lower tumor grades and enhanced survival of osteosarcoma affected cats.

The ability of tumor cell lines to produce tumors in immunodeficient laboratory animals is considered important for the characterization of neoplastic cell lines [18,20,21]. However, we consider the screened markers sufficient to confirm the origin of the newly produced cell lines as shown by other groups before [21,29,32]. However, comparative *in vivo* testing of the feline and canine osteosarcoma cell lines would, of course, add valuable data to their further experimental application. Therefore, their tumor formation capacity should be tested in future studies. 

## 5. Conclusions

We have confirmed the osteosarcoma origin of the newly developed cell lines. They broaden the available species for *in vitro* osteosarcoma research to a species that is known to have a much better survival prognosis than humans or dogs. Species-specific differences, like the distinct expression of the oncogene p53, could be helpful to elucidate the biological reasons for the differences in feline and canine osteosarcoma progression. This research might also open interesting experimental tools to test potential therapeutic applications *in vitro*.

## Figures and Tables

**Table 1:** Animals used for the creation of the new cell lines.

Sample	Resulting Cell Line	Species	Sex ^a^	Age	Breed	OS Subtype	Location
1033	COS_1033	Dog	F	7y 1m	Boxer	Teleangiectatic OS with giant cell participation	Lung metastasis
1077	FOS_1077	Cat	F	10y	Domestic shorthair	FibroblasticOS	Costa
1140	FOS_1140	Cat	M °	n/s	Domestic shorthair	Osteoblastic OS	Tibia
1186	COS_1186w COS_1186h	Dog	M °	11y 2m	Dachshund	Osteoblastic OS	Scapula
1189	COS_1189	Dog	M °	7y 3m	Mixed breed	Poorly defined OS/ signs of osteo-or synovialsarkom	Humerus
1220	COS_1220	Dog	F °	5y 3m	Boxer	Fibroblastic OS	Radius

**^a^** F = female, M = male, **°** = neutered; n/s = not specified.

**Table 2:** Details of procedures used for IHC staining.

Primary Antibody	Clone	Dilution	Antigen Retrieval		Source
Osteopontin	polyclonal	1:75	Tissue	30 min steamed in citric acid buffer pH 6.0	Biogenesis, Poole, UK
	rabbit		Cell pellet	2x 5 min microwaved in citric acid buffer pH 6.0	
Osteocalcin	polyclonal	1:400	Tissue	30 min steamed in citric acid buffer pH 6.0	Biogenesis
	rabbit		Cell pellet	2x 5 min microwaved in citric acid buffer pH 6.0	
Cytokeratin	monoclonal	1:250	Tissue	30 min steamed in Tris-EDTA buffer pH 9.0	Cell Marque, Rocklin, CA, USA
	mouse		Cell pellet	2x 5 min microwaved in Tris-EDTA buffer pH 9.0	
Vimentin	monoclonal	1:200	Tissue	20 min steamed in citric acid buffer pH 6.0	Dako, Glostrup, Denmark
	mouse		Cell pellet	2x 5 min microwaved in citric acid buffer pH 6.0	
Alkaline Phosphatase	polyclonal	Dog: 1:100	Tissue	30 min steamed in citric acid buffer pH 6.0	Genetex, Irvine, CA, USA
	rabbit	Cat: 1:250	Cell pellet	2x 5 min microwaved in citric acid buffer pH 6.0	
Osteonectin	polyclonal	1:1500	Tissue	none	Millipore, Billerica, MA, USA
	rabbit		Cell pellet	none	
p53	monoclonal	1:90	Tissue	3x 5 min microwaved in Tris-EDTA buffer pH 9.0	Enzo Life Sciences, Lausen, Switzerland
	mouse		Cell pellet	2x 5 min microwaved in Tris-EDTA buffer pH 9.0	

**Table 3:** PCR-primer and assay details.

Gene / Symbol	Species	NCBI Ref Nr (Dog/Cat)	Foward Primer	Reverse Primer	Length (bp)
Osteocalcin /	Dog	XM_547536.4	GCTGGTCCAGCAGATGCAA	CCCAGCCCAGAGTCCAGGTA	125
BGLAP	Cat	XM_003999711.3	GCCCGGCAGATGCAAAG	CCCTCCTGCTTGGACACGA	70
Osteopontin/	Dog	XM_003434023.2	ACTGACATTCCAGCAACCCAA	CACAAGTGATGTGAAGTCCTCCTCT	168
SPP1	Cat	XM_006930977.2	CAATTTTTCACCCCAGCTGTC	CACAAGTGATGTGAAGTCCTCCTCT	150
Osteonectin /	Dog	XM_005619272.1	CACCCTGGAAGGCACCAA	CGCAGAGGGAATTCAGTCAGC	108
SPARC	Cat	XM_003981374.3	CCAAGAAGGGCCACAAACTC	GGAATTCGGTCAGCTCGGA	87
Alkaline phosphatase /	Dog	XM_005617214.1	GGCCTGAACCTCATCGACAT	GCGGTTCCAGACGTAGTGAGA	72
ALPL	Cat	NM_001042563.1	GGACGGCCTGAACCTCG	GAGTTCGGT GCGGTTCCA	85

## Supplementary Materials

The following are available online at www.mdpi.com/2306-7381/3/2/9/s1, Table S1: Results of qualitative RT-PCR to determine the mRNA expression of ALPL, SPP1, SPARC and BGLAP; Table S2: Length of microsateites in dogs used to confirm the cell cultues origins; Table S3: Length of microsateites in cats used to confirm the cell cultues origins.
