# Supplementary material for: Establishment and Characterization of New Canine and Feline Osteosarcoma Primary Cell Lines
Source: Vet Sci. 2016 Jun 1;3(2):9. doi: 10.3390/vetsci3020009 (PMC5644629; doi:10.3390/vetsci3020009)
Supplement: Supplementary file 1 [file vetsci-03-00009-s001.pdf]

Supplementary Materials: Establishment and Characterization of New Canine and Feline Osteosarcoma Primary Cell Lines

Florian R. L. Meyer and Ingrid Walter

1. PCR

By means of PCR, the presence of mRNA of tissue unspecific alkaline phosphatase (*ALPL*) and osteonectin (*SPARC*) was detected positively in all tested feline and canine tissue and cell culture samples, except for the cell culture samples of COS_1220 which lost ALPL expression, mirroring the results obtained by immunohistochemistry. However, we failed to amplify osteocalcin (*BGLAP*) mRNA in feline and canine samples. Amplification of the genes in the individual tumour samples and the results of the corresponding melting curve analysis are depicted in Table S1.

Table S1. Results of qualitative RT-PCR to determine the mRNA expression of ALPL, SPP1, SPARC and BGLAP.

		ALPL		*SPP1*		*SPARC*		*BGLAP*	
		Amplicon	MC	Amplicon	MC	Amplicon	MC	Amplicon	MC
sample									
1033	Tumour	+	+	+	+	+	+	-	-
1033	CC	+	+	~	+	+	+	-	-
1077	Tumour	+	+	~	+	+	+	-	-
1077	CC	~	+	+	+	+	+	-	-
1140	Tumour	+	+	+	+	+	+	-	-
1140	CC	~	+	+	+	+	+	-	-
1186	Tumour	+	+	+	+	+	+	-	-
1186h	CC	+	+	+	+	+	+	-	-
1186w	CC	+	+	+	+	+	+	-	-
1189	Tumour	+	+	+	+	+	+	-	-

Table S1. *Cont.*

		ALPL		*SPP1*		*SPARC*		*BGLAP*	
		Amplicon	MC	Amplicon	MC	Amplicon	MC	Amplicon	MC
1189	CC	+	+	+	+	+	+	-	-
1220	Tumour	-	-	+	+	+	+	-	-
1220	CC	+	+	+	+	+	+	-	-
MC	melting curve								
+	positive result								
~	very late amplification signal								
-	negative result								

[+] Positive result; [~] very late amplification signal; [−] negative result.

2. DNA Fingerprint Assay

Microsatellite analysis of the established primary cell lines confirmed their origin from the corresponding tumor. In feline samples 16 markers were tested. All markers were identical between tumor tissue and cells in culture. In dogs, 18 markers were tested. COS_1033 and COS_1186h cells and tumor were completely identical in their marker setup, although one marker failed to amplify in the latter, both in tumor and in the cell line. COS_1220 showed one allele changed in a single marker, COS_1189 had alterations in two alleles. Three markers differed between COS_1186w and the tumor, although one marker failed to amplify in the tumor sample, but not in the cell line. A complete list of the obtained microsatellite data as first published in [1] is supplied in Table S2 and S3. These list can be helpful in future experiments to exclude cross contaminations.

Table S2. Length of microsateites in dogs used to confirm the cell cultues origins.

**Microsatelite** [a]	**1033 Tumour**	**COS_1033 Cell Culture**	**1186 Tumour**	**COS_1186h Cell Culture**	**COS_1186w Cell Culture**	**1189 Tumour**	**COS_1189 Cell Culture**	**1220 Tumour**	**COS_1220 Cell Culture**
AHT121:	102/102	102/102	-/-	-/-	98/102	106/112	106/116	102/102	102/102
AHT137:	131/135	131/135	135/137	135/137	135/137	131/139	131/139	131/131	131/131
AHTH171:	233/233	233/233	221/229	221/229	221/229	229/229	229/229	219/219	219/219
AHTH260:	238/244	238/244	238/244	238/244	238/244	246/250	246/250	244/244	244/244
AHTK211:	87/95	87/95	91/91	91/91	91/91	91/99	91/99	95/95	95/95
AHTK253:	288/292	288/292	288/292	288/292	288/292	290/292	286/290	288/288	288/288

Table S2. *Cont.*

Microsatelite [a]	1033 Tumour	COS_1033 Cell Culture	1186 Tumour	COS_1186h Cell Culture	COS_1186w Cell Culture	1189 Tumour	COS_1189 Cell Culture	1220 Tumour	COS_1220 Cell Culture
CXX279:	120/124	120/124	124/124	124/124	124/126	118/126	118/126	120/124	120/124
FH2054:	152/156	152/156	152/156	152/156	152/156	152/156	152/156	168/172	168/172
FH2848:	236/242	236/242	242/246	242/246	242/246	232/238	232/238	230/236	230/236
INRA21:	99/105	99/105	105/105	105/105	99/105	95/95	95/95	101/101	101/101
INU005:	124/124	124/124	122/126	122/126	122/126	110/124	110/124	124/132	124/132
INU030:	144/156	144/156	144/150	144/150	144/150	150/150	150/150	144/144	144/144
INU055:	210/222	210/222	210/218	210/218	210/218	210/210	210/210	210/222	210/222
REN162C04:	200/200	200/200	206/206	206/206	206/206	202/206	202/206	202/202	202/202
REN169D01:	216/216	216/216	212/216	212/216	212/216	210/212	210/212	214/216	216/216
REN169O18:	164/168	164/168	162/164	162/164	162/164	162/172	162/172	164/170	164/170
REN247M23:	272/272	272/272	268/268	268/268	268/268	268/272	268/272	268/272	268/272
REN54P11:	226/226	226/226	226/232	226/232	226/232	224/226	224/226	226/236	226/236

[a] Microsatelite names are based on the standards of the ISAG Comparison Test 2006.

Table S3. Length of microsateites in cats used to confirm the cell cultures origins.

Microsatelite [a]	1140 Tumour	FOS_1140 Cell Culture	1077 Tumour	FOS_1077 Cell Culture
FCA 069	103/107	103/107	105/109	105/109
FCA 075	132/134	132/134	126/134	126/134
FCA 105	197/205	197/205	201/203	201/203
FCA 149	124/124	124/124	124/130	124/130
FCA 220	214/214	214/214	212/214	212/214
FCA 229	168/168	168/168	168/168	168/168
FCA 310	138/138	138/138	128/130	128/130
FCA 441	155/155	155/155	155/159	155/159
FCA 678	190/192	190/192	190/192	190/192
FCA 005	148/148	148/148	144/148	144/148

Table S3. *Cont.*

Microsatelite [a]	1140 Tumour	FOS_1140 Cell Culture	1077 Tumour	FOS_1077 Cell Culture
FCA 026	146/146	146/146	156/158	156/158
FCA 201	145/155	145/155	143/161	143/161
FCA 224	160/160	160/160	160/160	160/160
FCA 293	187/187	187/187	187/187	187/187
FCA 453	188/200	188/200	188/196	188/196
FCA 649	138/138	138/138	126/140	126/140

[a] Microsatelite names are based on the standards of the ISAG Comparison Test 2006.

References

1. Meyer, F.R.L.; Steinborn, R.; Grausgruber, H.; Wolfesberger, B.; Walter, I. Expression of platelet-derived growth factor BB, erythropoietin and erythropoietin receptor in canine and feline osteosarcoma. *Vet. J.* **2015**, *206*, 67–74.

© 2016 by the authors; licensee MDPI, Basel, Switzerland. This article is an open access article distributed under the terms and conditions of the Creative Commons by Attribution (CC-BY) license (http://creativecommons.org/licenses/by/4.0/).
